# Cancer pain control in a Nigerian oncology clinic: treating the disease and not the patient

**DOI:** 10.11604/pamj.2021.40.104.25225

**Published:** 2021-10-15

**Authors:** Adedayo Olufemi Joseph, Omolola Salako, Adewunmi Alabi, Muhammadu Habeebu, Onyinye Balogun, Olubukola Ayodele, Opeyemi Mercy Awofeso, Adeniyi Adenipekun

**Affiliations:** 1 Department of Radiotherapy and Oncology, Lagos University Teaching Hospital, Lagos, Nigeria,; 2 Weill Cornell Medicine, New York-Presbyterian Hospital, Department of Radiation Oncology, New York, United State of America,; 3 Princess Margaret Cancer Centre, Toronto, Canada,; 4 Lagos University Teaching Hospital, Lagos, Nigeria,; 5 University College Hospital, Department of Radiation Oncology, Ibadan, Nigeria

**Keywords:** Cancer pain, cancer management, low-to-middle-income country

## Abstract

**Introduction:**

inadequate pain control negatively impacts the quality of life of patients with cancer while potentially affecting the outcome. Proper pain evaluation and management are therefore considered an important treatment goal. This study assessed the prevalence of pain, the prescribing patterns, and the efficacy of pain control measures in cancer patients at the Radiation Oncology Unit of the Lagos University Teaching Hospital, Lagos.

**Methods:**

this was a longitudinal study design recruiting adults attending outpatient clinics. Participants were assessed at initial contact and again following six weeks using the Universal Pain Assessment Tool developed by the UCLA Department of Anaesthesiology.

**Results:**

among the patients reviewed, 34.0% (118 of 347) were at the clinic, referred for initial assessment following primary diagnosis. All respondents had solid tumours; the most common was breast cancer. The prevalence of pain at initial assessment was 85.9% (298 of 347), with over half of respondents, 74.5% (222 of 347) characterising their pain as moderate to severe. Over a quarter, 28.9% (100 of 347) of patients were not asked about their pain by attending physicians, and none of the patients had a pain assessment tool used during evaluation. In 14.4% (43 of 298) of patients, no intervention was received despite the presence of pain. At six weeks review, 31.5% (94 of 298) of patients had obtained no pain relief despite instituted measures.

**Conclusion:**

under-treatment of cancer pain remains a significant weak link in cancer care in (Low-to-middle-income country) LMICs like Nigeria, with a significant contributor being physician under-evaluation and under-treatment of pain. To ensure pain eradication, the treatment process must begin with a thorough evaluation of the patient's pain, an explicit pain control goal and regular reevaluation.

## Introduction

The history of pain in human affliction is a long and dreadful one. The word “pain” itself originated from the Latin word “*Poine*”, meaning penalty. It was believed that the Greek goddess of revenge, *Poine*, was sent to punish the mortals who had angered the gods [1]. From the dawn of the ages, many cultures have viewed pain as punishment for wrongdoing. This attitude persists in our societal subconscious to this day [2]. Even worse, pain is subconsciously accepted as an unavoidable aspect of any illness, especially one as “serious” as cancer. As such, if pain control is to be properly executed in cancer management, both patients and healthcare providers must overcome this subconscious barrier, which subtly encourages them to “ignore” or “accept” pain.

In 1811, French writer and courtier Frances Burney underwent a mastectomy for suspected breast cancer without any anaesthesia or analgesia. She documented her ordeal in a very graphic and vivid letter to her sister, thus producing one of the earliest and most detailed, albeit gruesome, accounts of a mastectomy [3,4]. With centuries of advancement in medicine and cancer care, one would hope that such agonising and torturous pain caused either by disease or therapy would be firmly confined to the coffers of history. This, unfortunately, is not the case. Patients living with cancer all over the world endure immeasurable pain, day in and day out [5]. This pain is brought on either as the direct effect of tumour infiltration, investigational and therapeutic interventions, or non-cancer-related comorbidities, all of which the physician has the responsibility to address as part of holistic patient care [6]. Especially in low and middle-income counties like Nigeria, a cancer diagnosis is often unfortunately associated with a sentence of pain, suffering, and death [7].

Pain is one of the most common symptoms experienced by people living with cancer [8]. An estimated 30% of patients have pain as their first presenting symptom, while in the last few weeks of life, especially with the advancement of cancer stage, the prevalence of moderate to severe pain rises to as high as 70% [9]. It may seem to the casual observer that pain would be an expected and unavoidable part of the cancer journey. However, it is crucial for the clinician to understand that pain is a complex symptom that affects most aspects of the patient's life, from physical functioning to daily living; as well as psychological, emotional and social status; and if properly managed, can be avoided or at least controlled [10]. Under-treatment of pain has profound and varied effects on the patient, family and society at large [11]. Although significant milestones have been made in understanding the physiology of pain, there remains a deficiency in the complete control of pain; due to a dynamic combination of socio-cultural, educational, economic, religious, legal, and political factors [12]. In many cases, it is essential for the healthcare provider first to attempt to alleviate the patient's physical pain with cancer, as this might significantly impact other aspects of the physical, social and mental health of the patient.

The obstacles to proper management of pain in resource-constrained settings like Nigeria are numerous and inflected by factors peculiar to economically challenged countries [13]. A considerable proportion of patients with cancer in Nigeria present with an advanced and often incurable disease, situations in which pain is almost always present, and pain control becomes a priority [14]. Another complication is the relative inaccessibility or unavailability of essential pain control medications [15]. This combination of high proportions of patients presenting with advanced disease, and the deficiency of crucial pain control measures, culminates in a desperate situation for the patient with cancer in Nigeria. Furthermore, there is a suspected under-exploration of pain control in oncology care, particularly in countries like Nigeria. This area remains poorly documented in formal literature and under-explored for clinical medicine, research and policy. This study, therefore, assessed pain control in patients with cancer attending outpatient clinics at the Radiotherapy and Oncology Department of the largest and oldest tertiary healthcare facility in Lagos, Nigeria; intending to outline the current practices and the opportunities for improvement to ensure a better quality of life for patients living with cancer in Nigeria.

## Methods

**Study design**: this project employed a longitudinal study design to recruit adult patients with cancer seen at the Radiotherapy and Oncology clinic of the Lagos University Teaching Hospital (LUTH).

**Study setting**: Nigeria is the most populous country in Africa, with an estimated population of 200 million and accounts for over 100,000 new cancer cases every year. Lagos is the most economically advanced and populated city in Nigeria, and LUTH is one of the largest teaching hospitals in Nigeria, located in the former capital, Lagos state, providing care for patients with cancer referred from all over the country and neighbouring states. The hospital has over 700 beds; and is equipped with medical care infrastructure, training programs, and research infrastructure [16]. This study was conducted at the Radiotherapy and Oncology clinic of the Lagos University Teaching Hospital (LUTH) [16]. The Radiotherapy and Oncology Department was the first formal Oncology department in the country; established following a cabinet decision in April 1961 [16].

**Study participants**: study participants included all adult patients with histologically confirmed cancer who presented to the outpatient clinics for first treatment following diagnosis or follow up treatments and were willing to participate in the study. Patients in remission or cancer survivors who attended the clinics for follow up were excluded from the study. Patients with recurring cancers and second cancers were also excluded. Ethical approval for this research was obtained from the Health Research and Ethics Committee of the LUTH before the commencement of the study, and each patient was educated on the study protocol, possible benefits, risks and voluntary nature of the survey before recruitment. Written informed consent was given and signed by all patients before data collection.

**Study variables and data sources**: in all participants, information was collected with the aid of a pre-designed data collection tool that recorded parameters such as sociodemographic and clinical characteristics of the patients. At this initial review, pain assessment and severity were determined using the Universal Pain Assessment Tool developed by the UCLA Department of Anaesthesiology [17]. Each patient was then followed up for six weeks and interviewed again. At follow up review, each patient was interviewed, and parameters including pain assessment, severity, patterns of pain management by their attending physician were recorded.

**Bias**: potential sources of bias for this study were from selection bias of patients in later stages of their diseases, as such with a higher likelihood of pain. This was controlled for, in the study design by the use of the same criteria for recruitment for all adult patients with cancer seen at the clinic regardless of the disease stage or clinical condition. Another anticipated source of bias was from recall by patients regarding their pain assessment. This was mitigated in the design phase by a cross-reference of patients´ reports with physician documentation of pain.

**Study size**: the study was carried out over five months between July and December 2016. All patients seen in the clinics who meet the study criteria and consented to the study were included until the study duration elapsed. A total of 358 patients were enrolled.

**Quantitative variables and statistical methods**: the data were analysed using the IBM SPSS Statistics for Windows (Version 22.0. Armonk, NY: IBM Corp). Descriptive analysis was used in reporting variables such as participants' sociodemographic and clinical characteristics, the prevalence of pain and the pattern of analgesics prescribed and reported using frequency tables and charts. A comparison was made between the prevalence and severity of pain at the initial and follow up review. Factors including gender, stage at diagnosis and stage at care were associated with the presence and severity of pain to check for significance. Missing data were excluded from analyses. A P-value of <0.05 was taken as significant.

## Results

**Participants**: a total of 358 clinic attendees were asked to fill an interviewer-administered questionnaire. Of these, 347 were adequately completed and subsequently analysed, giving a response rate of 95.7%.

**Figure 1:**
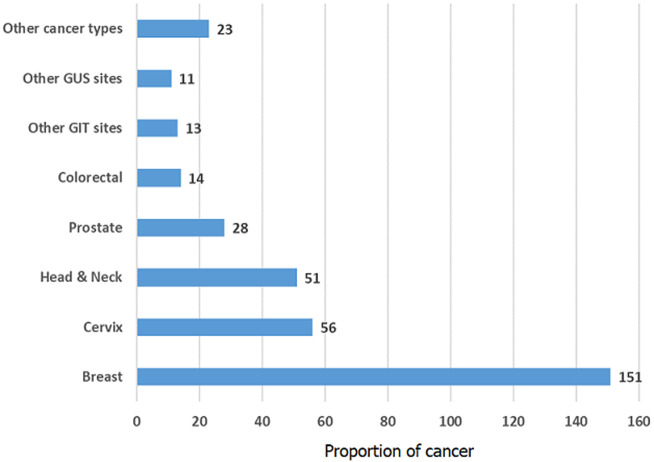
primary tumour site in patients with cancer seen at the Radiotherapy and Oncology Unit of LUTH between July and December, 2016 (N=347)

**Descriptive data**: female respondents outnumbered males at a ratio of almost 1.5: 1, with the majority of respondents falling within the World Health Organization definition of middle- (35-54 years) and older- (55-74 years) aged adults. Of the respondents, 34.0% (118 of 347) were new patients attending the clinic for the first time for treatment following their diagnosis, while 66.0% (229 of 347) were previously registered patients who had been seen on least one previous occasion for treatment. All respondents had solid tumour types, with the most prevalent primary cancer sites been Breast in 43.5% (151 of 347), Cervix in 16.1% (56 of 347), Head and Neck in 14.7% (51 of 347); and Prostate cancers in 8.1% (28 of 347) patients (Figure 1). The majority of the respondents were in advanced cancer stages at diagnosis: locally advanced 59.4% (206 of 347) metastatic 14.1% (49 of 347.) Respondents with early-stage cancers made up 27.1% (94 of 347) of the study group.

**Outcome data**: using the universal pain assessment tool, the prevalence of pain at first assessment was 85.9% (298 of 347) based on a score of 1 or more on the Universal Pain Assessment Tool, 14.1% (49 of 347) of patients had pain scores of 0 on the Universal Pain Assessment Tool, representing no pain at all. In patient with pain, 25.5% (76 of 298) had mild pain (1-3), 56.7% (169 of 298) of patients had pain scores in the moderate range of the Universal Pain Assessment Tool (4-7) and 17.8% (53 of 298) described pain as severe (8-10). The self-reported primary cause of pain was the cancer as seen in 90.9% (271 of 298) of patients. Additional sources of pain identified were medical tests and investigations such as blood tests or biopsies and treatment modalities, including chemo- or radiation- therapy. Non-cancer related causes of pain were reported in 8.7% (26 of 298) of patients. These included pre-and co-existing conditions such as Arthritis, Diabetic Neuropathy and Migraine disorders (Table 1).

**Table 1:** the clinical history of patients with cancer recruited from the Radiotherapy and Oncology Department of LUTH from July to December, 2016 (N=347)

Variable	n[%]
**Pre-existing pain condition** (n=347)	
Arthritis	46[13.2]
Diabetic neuropathy	3[0.9]
Migraine disorder	1[0.3]
**Causes of Pain** (n=298)	
Primary cancer	271[90.9]
Treatment e.g. Chemo, RT	94[31.5]
Investigations, e.g. Biopsy, blood tests	37[12.4]
Other non-cancer causes [comorbidities]	26[8.7]
**Assessment of pain by physicians** (n=347)	
Yes	247[71.1]
No	100[28.9]
**Pain relief at follow-up appointment(**n=231**)**	
Worsened	46[20.0]
Improved	139[59.3]
No change	48[20.8]

In 14.4% (43 of 298) of patients with pain, no pain-directed treatment was prescribed. The only strong oral opioid available at the oncology pharmacy at the time of the study was Morphine. In 79.5% of patients with pain (237 of 298) of the patients were offered oral analgesic medications. In general, Non-Steroidal Anti-Inflammatory Drugs (NSAIDs) were the most common analgesic prescribed to patients with pain as seen in 67.8% (202 of 298) of patients, Oral NSAIDs were prescribed three times more than oral Opioids. Parenteral analgesia was prescribed to 12.1% (36 of 298) of patients, of which parenteral NSAIDs was given in 6.4% (19 of 298) and Opioids in 5.7% (17 of 298) of patients with pain in almost equal proportions (Table 2). In 6.4% (19 of 298), Morphine was prescribed often prescribed alone or in combination with other analgesic modalities (Table 2). Acetaminophen was prescribed in oral form in 14.1% of patients with pain (42 of 298) but no parenteral acetaminophen was prescribed. Topical analgesia was only prescribed in 1.0 (3 of 298) of patients (Table 2). In 23.2% (69 of 298) respondents in pain, palliative radiation therapy to the pain site was offered, with an additional prescription for adjuvant medications. Interventional methods such as surgical nerve blocks were not prescribed for any of the respondents. About 7% (21 of 298) of respondents were counselled to employ rest as a pain control modality. Adjuvant medications prescribed to 53.0% of patients (158 of 298); consisting of Corticosteroids (Dexamethasone, Prednisone), Bisphosphonates (predominantly Zoledronic acid), Benzodiazepines (Bromazepam), and Tricyclic Antidepressants (Amitriptyline). Dexamethasone was the most prescribed steroid (Table 2).

**Table 2:** pattern of pain medications prescribed to patients with cancer at the radiotherapy and oncology unit of LUTH between July and December, 2016 (N=298)

Variable (n=298)	n[%]
**Oral Analgesic**	237[79.5]
Oral NSAIDs e.g. Ibuprofen	183[61.4]
Oral Opioids	54[18.1]
Weak e.g. Dihydrocodeine	35[11.7]
Strong e.g. Morphine	19[6.4]
Oral acetaminophen	42[14.1]
**Parenteral Analgesic**	36[12.1
Parenteral Opioids	17[5.7]
Weak e.g. Tramadol	1[0.3]
Strong e.g. Pentazocine	16[5.4]
Parenteral NSAIDs e.g. Diclofenac	19[6.4]
**Topical Analgesic**	3[1.0]
**Palliative radiation therapy**	69[23.2]
**Other e.g. rest**	21[7.0]
**Steroids**	158[53.0]
**Bisphosphonates**	81[27.2]
**Benzodiazepines**	13[4.4]
**Tricyclic antidepressants**	1[0.3]
**Interventional**	0[0.0]
**No Treatment Given**	43[14.4]

* Multiple responses allowed

Over a quarter, 28.9% (100 of 347) of the patients seen reported that their attending physician did not ask if they were in pain. Even in patients who were asked about their pain, they uniformly stated that their attending physician used no pain assessment tool of any kind or any measure of pain severity quantification to assess their pain during clinic consultations, treatments or follow-ups. At follow up review at 6 weeks, 77.5% (231 of 298) of the patients with pain at initial review were re-interviewed, 22.4% (67 of 298) were lost to follow-up. Of the patients with pain at the initial review. 46.0% (137 of 298) had obtained relief from the instituted pain relief measures, indicated by a lower pain score on the Universal Pain Assessment Tool, (94, 31.5%) of the respondents had not obtained pain relief; indicated by unchanged or higher pain scores when compared to the initial interview. There was a statistically significant association between the presence and severity of pain and the stage diagnosis of cancer and stage at treatment. There was a statistically significant association between the gender of the patient and the perception of pain severity but not the presence or absence of pain (Table 3).

**Table 3:** factors associated with the presence and severity of pain in patients with cancer seen at the Radiotherapy and Oncology unit of LUTH between July and December, 2016 (N=347)

Variable	Presence of pain			Test statistic P-value
	Yes	No		
**Gender**				
Female	176	31		X^2^=0.16 P=0.689
Male	122	18		
**Stage at care**				
First treatment	80	39		X^2^=20.13 P=<**0.001**
Follow-up treatment	210	30		
**Stage at diagnosis**				
Early	35	59		
Locally advanced	9	196		X^2^=57.96 P=**0.000**
Metastatic	5	43		
**Pain Severity**	**Mild**	**Moderate**	**Severe**	
**Gender**				
Female	59	85	14	X^2^=34.05 P=**0.001**
Male	17	84	39	
**Stage at care**				
First treatment	56	20	12	X^2^=97.84 P=**0.001**
Follow-up treatment	20	149	41	
**Stage at diagnosis**				
Early	43	14	5	
Locally advanced	25	148	21	X^2^=156.91 P=**0.001**
Metastatic	8	7	27	

## Discussion

The International Association for the Study of Pain (IASP) defines pain as an unpleasant sensory and emotional experience associated with actual or potential tissue damage or described in terms of such damage [18]. It further states that pain is always subjective and that every individual learns the application of the word through experiences related to injury in early life [19]. Pain management remains a crucial aspect of the management of patients living with cancer globally; this is especially important in the face of the increasing numbers of cases of people with cancer and living with cancer-related morbidities. Especially in developing countries like Nigeria, there requires a shift from the traditional focus on only the eradication of the disease to a management plan which involves an interdisciplinary model of healthcare providers who treat the whole person, including the physical, emotional, and mental health with a comprehensive pain eradication or management plan [20].

In this study, the prevalence of pain was at 85.9% (298 of 347); this finding isn't far removed from a similar study carried out in cancer patients in at the University College Hospital, Ibadan Nigeria where the prevalence of pain was 73.8% [21]. It was also reported that 17.8% (53 of 298) of respondents in this study had severe pain as at the time of the first review, while 56.7% (169 of 298) of patients rated their pain between 4 and 7 (moderate pain). Another study, a one-year, one-thousand-patient, Eastern Cooperative Oncology Group (ECOG) study carried out by Cleeland and colleagues found more than half of the study subjects rated their pain as moderate to severe [22]. Non-cancer-related pain represented was the primary cause of pain in 8.7% (26 of 298) of patients and were due to comorbid conditions such as arthritis, diabetic neuropathy or migraine disorder.

Over a quarter, 28.9% (100 of 347) of the patients seen in this study were not asked about their pain by the physician that attended to them. This means that for every ten patients attended to, up to 3 walked away without being asked by their doctor if they were having any pain. All respondents uniformly reported that the attending physician had not used any tool to assess their pain's intensity. This finding is in stark contrast to a study in the US which found that almost all 300 of the centres surveyed used some form of the pain assessment tool, most commonly the Numeric rating scale, with at least half of them including adequate pain control as an indication of quality assurance [23]. Perhaps the inclusion of pain management and pain assessment tools in Oncology training curriculums and medical training programs in developing countries like Nigeria would improve the Oncologist's awareness, understanding and ability to evaluate for and manage the patients' pain. In addition, routine use of pain assessment tools (including fast and easy-to-administer tools like the Numerical Rating Scale, Visual Analog Scale etc.) to determine pain intensity should improve the evaluation and, consequently, the management and monitoring of pain in patients with cancer. Every clinic visit documentation should include an evaluation of the presence or absence of pain and the severity as expressed by the patient. A pain assessment tool should ensure an objective way of determining whether there is an improvement in the patient's pain and the efficacy of instituted pain control measures.

Only 6.4% (19 of 298) of the patients with pain received a strong opioid analgesic, despite the finding of moderate pain in 56.7% (169 of 298) and severe pain in 17.8% (53 of 298) of patients with pain reviewed. This disparity may be used as a marker of inadequate pain control as opioid analgesia is the gold standard for controlling moderate to severe cancer pain [24]. Other strong opioids indicated for the relief of cancer pain, such as Oxycodone, Buprenorphine and Fentanyl, were not available at the Oncology pharmacy during the period of the study. Therefore, patients attending the clinic were restricted to the only available strong opioid-morphine. Developing countries have long been plagued with this limitation of analgesic medications [25]. Size and Soyannwo in 1998 documented opioid legislation and lack of access in developing countries as a major barrier to cancer pain control; unfortunately, the situation remains only slightly improved even now [26].

Current cancer pain management guidelines encompass both pharmacologic and non-pharmacologic approaches such as psychotherapy and interventional modalities like surgical and ablative techniques [27]. Respondents in this study did not receive any recommendations for any kind of interventional therapy. Analgesic medication should be instituted and prescribed using the World Health Organization analgesic ladder, and adjuvant medications should be used when indicated. Opiophobia amongst Oncologists must be addressed as an integral part of oncology training to ensure that patients receive more effective analgesia when indicated. Legislation to improve opioid access through importation or production of presently unavailable opioid medications need also be instituted to enhance patient access. Inclusion of these medications on the National Health Insurance Scheme or subsidisation by the government may also benefit this regard.

Alternative pain control therapies should be explored and employed more often. Cognitive-behavioural therapies and coping mechanisms may be as effective in managing a cancer patient's pain as medication. More research into these areas would serve to improve knowledge and possibly, patient quality of life. Pain control should be included as a stand-alone treatment goal at the start of cancer treatment, regardless of treatment intent (i.e. radical or palliative). This would serve to encourage physicians to view the alleviation of pain as a priority instead of an addition. The authors recognise that a possible limitation of the study is the possible recall bias from the patients who were asked about the physician evaluation of their pain. However, case records were reviewed to mitigate this bias for documentation of pain evaluation and management to corroborate patient accounts.

## Conclusion

Pain is arguably the most common symptom experienced by cancer patients and encountered by Oncologists. How unfortunate then that despite several published guidelines for assessing and managing pain, undertreatment of pain continues to plague oncology practice in Nigeria. This study reflects a high pain prevalence among adult patients with cancer attending a Nigerian clinic, with accompanying under-evaluation and under-treatment. This physicians´ under-evaluation and undertreatment of pain coupled with the unavailability of gold standard medications for pain treatment all contribute to the persistence of poorly controlled pain in patients living with cancer in this clinic and arguably other oncology clinics in Nigeria. The physician must remember always to treat the patient, not just the disease. Pain control must be regarded as a fundamental part of the individual's well-being and management goal, without which there can be no conclusion of declaring that patient as “treated”. We need continued pieces of literature, clinical discussions and tools, and advocacy for implementation of findings to bring this crucial area into care and justify its importance so that patients are treated comprehensively as they face cancer and its disabling impact.

### What is known about this topic

- Pain is a common but often avoidable or manageable aspect of the cancer journey;
- Proper pain control significantly impacts the quality of life of the patient with cancer.

### What this study adds

- Pain management of patients with cancer in Lagos is suboptimal;
- A major contributor to suboptimal pain management in patients with cancer is the physician under-evaluation of pain during clinic visits.
